# Muscle synergies demonstrate only minimal changes after treatment in cerebral palsy

**DOI:** 10.1186/s12984-019-0502-3

**Published:** 2019-03-29

**Authors:** Benjamin R. Shuman, Marije Goudriaan, Kaat Desloovere, Michael H. Schwartz, Katherine M. Steele

**Affiliations:** 1 Department of Mechanical Engineering, University of Washington, Stevens Way, Box 352600, Seattle, WA 98195 USA; 2 Department of Human Movement Sciences, VU university, Amsterdam, the Netherlands; 3 Department of Rehabilitation Science, KU Leuven, Leuven, Belgium; 4 Clinical Motion Analysis Laboratory, University Hospitals Leuven Campus Pellenberg, Pellenberg, Belgium; 5 James R. Gage Center for Gait & Motion Analysis, Gillette Children’s Specialty Healthcare, St. Paul, MN USA; 6 Department of Orthopaedic Surgery, University of Minnesota, Minneapolis, MN USA

**Keywords:** CP (cerebral palsy), Gait, Motor disorders, Muscle synergy, Electromyography, Neurological rehabilitation, Motor control, Synergy plasticity

## Abstract

**Background:**

Children with cerebral palsy (CP) have altered synergies compared to typically-developing peers, reflecting different neuromuscular control strategies used to move. While these children receive a variety of treatments to improve gait, whether synergies change after treatment, or are associated with treatment outcomes, remains unknown.

**Methods:**

We evaluated synergies for 147 children with CP before and after three common treatments: botulinum toxin type-A injection (*n* = 52), selective dorsal rhizotomy (*n* = 38), and multi-level orthopaedic surgery (*n* = 57). Changes in synergy complexity were measured by the number of synergies required to explain > 90% of the total variance in electromyography data and total variance accounted for by one synergy. Synergy weights and activations before and after treatment were compared using the cosine similarity relative to average synergies of 31 typically-developing (TD) peers.

**Results:**

There were minimal changes in synergies after treatment despite changes in walking patterns. Number of synergies did not change significantly for any treatment group. Total variance accounted for by one synergy increased (i.e., moved further from TD peers) after botulinum toxin type-A injection (1.3%) and selective dorsal rhizotomy (1.9%), but the change was small. Synergy weights did not change for any treatment group (average 0.001 ± 0.10), but synergy activations after selective dorsal rhizotomy did change and were less similar to TD peers (− 0.03 ± 0.07). Only changes in synergy activations were associated with changes in gait kinematics or walking speed after treatment. Children with synergy activations more similar to TD peers after treatment had greater improvements in gait.

**Conclusions:**

While many of these children received significant surgical procedures and prolonged rehabilitation, the minimal changes in synergies after treatment highlight the challenges in altering neuromuscular control in CP. Development of treatment strategies that directly target impaired control or are optimized to an individual’s unique control may be required to improve walking function.

## Background

Cerebral palsy (CP) is caused by an injury to the brain at or near the time of birth [1]. Individuals with CP have impaired control and coordination of their muscles, as well as a variety of secondary musculoskeletal impairments. Muscle synergies have recently been used to evaluate and quantify impaired motor control in CP. Synergies are calculated from electromyography (EMG) data to identify weighted groups of muscles commonly activated together. Children with CP have altered synergies during gait compared to typically-developing (TD) peers [2–7], similar to other clinical populations such as stroke [8–12], spinal cord injury [13–15], and Parkinson’s Disease [16, 17]. Fewer synergies are required to describe muscle recruitment during dynamic tasks in CP, which is thought to contribute to impaired movement [2, 12, 15].

Recent research has suggested that synergies measured prior to treatment are associated with changes in gait after treatment in CP [18–20]. A summary measure of synergy complexity, the dynamic motor control index during walking (Walk-DMC), measured before treatment, has been shown to be associated with changes in gait kinematics and walking speed at two clinical centers [18, 20]. Children with greater synergy complexity, meaning synergies more similar to TD peers, are more likely to have improvements in gait kinematics and walking speed after single-event multi-level orthopaedic surgery (SEMLS), selective dorsal rhizotomy (SDR), or botulinum toxin injections type-A (BTA). While this research has suggested that synergy-based measures may be useful for treatment planning, the impact of these treatments on synergies is an open question. Researchers have proposed that treatments that can modify synergies may be clinically useful and contribute to improvements in movement [21–23]. However, whether or to what extent treatments can alter synergies or how those changes relate to functional outcomes remains unknown.

Few prior investigations have examined whether synergies can be altered as a result of an treatment [24–26]. Focusing mainly on rehabilitation after stroke, these studies have found mixed results, but have demonstrated that treatments have the potential to alter muscle synergies. For example, after rehabilitation therapies in stroke, synergy complexity has been found to increase [24], or have minimal changes [25], while in Parkinson’s, synergy complexity has been found to decrease [26]. All of these studies found some reorganization of synergy weights and/or timings after treatment [24–26]. In CP, preliminary research has suggested that there are minimal changes in synergies following treatment. For example, van der Krogt et al. (2016) reported a slight reduction in synergy complexity (i.e., further from TD peers) following BTA, while Oudenhoven et al. (2016) and Loma-Ossorio Garcia (2015) reported little change in synergy complexity following SDR or SEMLS, respectively [27–29]. Changes in synergy weights or activations after treatment have not been examined in CP.

The aim of this research was to examine whether common treatments in CP result in changes to synergy complexity, weights, or activations. Individuals with CP present a compelling population in which to examine changes in synergies due to the variety of treatments, often including extensive rehabilitation. Treatments, such as SDR, target the nervous system directly, while orthopedic surgery largely targets the musculoskeletal system. Injections of BTA provide short-term changes in muscle activity versus the long-term neuromuscular changes from SEMLS or SDR. If synergies change after SEMLS, SDR, or BTA, this could suggest that intensive rehabilitation or targeted treatments may be able to modify impaired control in children with CP. In contrast, if treatments do not alter synergies, these results could suggest that motor control is relatively fixed in CP.

## Methods

### Participants

We retrospectively analyzed pre- and post-treatment EMG and kinematic data collected at UZ Pellenberg, Belgium, during clinical motion analysis for 147 children with spastic CP (Table 1). The children with CP were distributed between three treatment groups: BTA, SDR, and SEMLS. All children were in Gross Motor Function Classification System (GMFCS) Levels I-III. We also evaluated gait for 31 typically-developing (TD) children for comparison to the children with CP. Apart from two TD children who had one walking trial, all participants completed a minimum of two barefoot, self-selected speed walking trials. Some of the children with more severe impairments (GMFCS Level III) walked with support, either from a therapist or assistive device. Marker trajectories were tracked using a 10 to 15 camera VICON system (Nexus 1.8.4, Vicon-UK, Oxford, UK), sampled at 100 Hz. Joint kinematics were calculated using the marker set of the lower limb Plug-in-Gait (PiG) model.

**Table 1:** Participant demographics

Treatment	N	GMFCS	Age	Gender	Height	Mass
		I/II/III	y + mo	F:M	meters	kg
BTA	52	18/19/15	6 + 10 (2 + 11)	19:33	1.15 (0.16)	21.3 (8.7)
SDR	38	11/23/4	9 + 4 (2 + 0)	20:18	1.33 (0.10)	29.7 (6.2)
SEMLS	57	20/17/20	12 + 2 (3 + 1)	23:34	1.45 (0.16)	39.3 (14.8)
TD	31	–	9 + 3 (2 + 9)	17:14	1.38 (0.17)	33.8 (13.3)

NOTE. Values are average (1 SD) or as otherwise indicated

*N* Number of Participants, *GMFCS* Gross Motor Function Classification System, *y + mo* Years + Months, *F* Female, *M* Male, *BTA* Botulinum Toxin Type-A Injection,

*SDR* Selective Dorsal Rhizotomy, *SEMLS* Single Event Multi-Level Orthopaedic Surgery, *TD* Typically-Developing Children

### Electromyography

Surface EMG data (Wave Wireless EMG, Cometa, Bareggio, Italy) were collected at either 1000 Hz or 1500 Hz from eight muscles bilaterally (gluteus medius, rectus femoris, vastus lateralis, medial hamstrings, lateral hamstrings, tibialis anterior, gastrocnemius, and soleus) during clinical gait analysis. Raw EMG data were band-pass filtered between 20 and 500 Hz upon collection. EMG data were analyzed from the more impaired side, when clinically indicated (*n* = 33, hemiplegic children and diplegic children with a more impaired side), and otherwise from a random side for each child (*n* = 114, diplegic children). All trials with EMG data (range = 1 to 12 trials, IQR = 2 to 4 trials) were concatenated within a session (pre- or post-treatment) for each child to maximize the number of steps for analysis [30]. For each trial, we excluded the first and last 10% of the EMG data at the beginning and end of each trial to avoid periods of acceleration and deceleration [31]. A linear envelope was calculated for each muscle using the following EMG data processing steps: high-pass filtered at 20 Hz, rectified, low-pass filtered at 10 Hz, amplitude scaled to the muscle’s maximum activation across all trials from a session, and down-sampled to 100 Hz [31].

### Synergy analysis

We calculated synergies using weighted non-negative matrix factorization (WNMF) in Matlab (MathWorks Inc., Natick, Massachusetts, United States) using the Matrix Factorization Toolbox [32, 33]. As with traditional non-negative matrix factorization (NMF), WNMF finds a set of synergy weights (W_mxn_) and activations (C_nxt_) such that EMG = W × C + error, where, *m* is the number of muscles (8 in this study), *t* is the number of EMG data points, and *n* is the number of synergies. WNMF differs from traditional implementations of NMF in that it assigns each data sample a weight ( 1= EMG present, 0 = EMG absent). We selected the WNMF algorithm to accommodate our clinical data set, which contained poor or missing EMG channels for 15% of all trials. For example, in some individuals there was missing data from one muscle and between trials the electrode was switched with another muscle’s electrode such that EMG data for each muscle was recorded in at least one trial. In each concatenated session, all eight muscles were recorded in at least one trial, ensuring that each muscle was represented in the synergy outputs for each child. The following settings were used for WNMF: 50 replicates, 1000 maximum iterations, 1 × 10^− 4^ minimum threshold for convergence, and 1 × 10^− 6^ threshold for completion.

### Synergy complexity

To evaluate synergy complexity, the total variance accounted for by *n* synergies (tVAF_n_) was calculated as [34, 35]:

1 $$ {\boldsymbol{t}\boldsymbol{VAF}}_{\boldsymbol{n}}=\left(\mathbf{1}-\frac{\left[{\sum}_{\boldsymbol{j}}^{\boldsymbol{t}}{\sum}_{\boldsymbol{i}}^{\boldsymbol{m}}{\left({\boldsymbol{error}}_{\boldsymbol{i},\boldsymbol{j}}\right)}^{\mathbf{2}}\right]}{\left[{\sum}_{\boldsymbol{j}}^{\boldsymbol{t}}{\sum}_{\boldsymbol{i}}^{\boldsymbol{m}}{\left({\boldsymbol{EMG}}_{\boldsymbol{i},\boldsymbol{j}}\right)}^{\mathbf{2}}\right]}\right)\times \mathbf{100}\% $$

We calculated the number of synergies required for tVAF_n_ > 90% (N_90_). Number of synergies has been used extensively to evaluate synergies in both unimpaired individuals and clinical populations [8, 24, 36], with prior research indicating that children with CP require fewer synergies than TD peers [2, 5].

The total variance accounted for by a single synergy solution (tVAF_1_) provides a summary measure of synergy complexity that has been shown to be related to function and treatment outcomes in CP [18, 20]. To contextualize the magnitude of changes in tVAF_1_ relative to TD peers and compare to prior research, the Dynamic Motor Control Index during Walking (Walk-DMC) was calculated as a scaled z-score of tVAF_1_, where tVAF_AVG_ and tVAF_SD_ are the average and standard deviation of tVAF_1_ of the TD individuals. Walk-DMC is scaled such that the average score is 100 for TD peers with a 10-point change representing one standard deviation of the TD group.

2 $$ walk- DMC=100+10\left[\frac{tVAF_{AVG}-{tVAF}_1}{tVAF_{SD}}\right] $$

We evaluated whether either measure of synergy complexity, N_90_ or tVAF_1_, changed after treatment. We also evaluated whether synergy complexity was similar between groups pre-treatment.

### Synergy composition

We also examined whether synergy weights or activations changed after treatment [24]. To provide context, we compared synergy weights and activations to TD peers. For the TD group, four synergies explained over 90% of the variance in EMG data for 81% of individuals (19% required five synergies). Thus, the average synergy weights and activations for four synergies was calculated for the TD group to define the archetype synergies. The archetype synergies had similar weights as previously published analyses of TD adults and children: C1 consisted primarily of extensor activity (gluteus medius, rectus femoris, and vastus lateralis); C2 consisted primarily of the plantarflexors (gastrocnemius and soleus); C3 consisted primarily of the tibialis anterior and rectus femoris; and C4 consisted primarily of the medial and lateral hamstrings [4, 8, 37]. We calculated the four-synergy solution for each child with CP and computed the cosine similarity (un-centered correlation coefficient) with the archetype synergy weights and activations. As both synergy weights and activations from WNMF are purely positive, cosine similarity constrains the correlation coefficient between 0 and 1, where a higher similarity indicates synergies that are more similar to TD peers. We evaluated whether similarity to TD peers changed after treatment, comparing the similarity of synergy weights and activations to the TD archetypes before and after each treatment [38]. We also evaluated whether the similarity of synergies to the TD archetypes differed between treatment groups pre-treatment.

### Changes in gait

In addition to EMG data, kinematic data from the clinical gait analyses were used to assess changes in gait post-treatment using two measures: walking speed and the gait deviation index (GDI). Walking speed was calculated from the average fore-aft velocity of the sacral marker for each trial and non-dimensionalized [39] as *walking speed* (*m*/*s*)/ √ (*leg length* (*m*) ∗ *gravity*(*m*/*s* ^ 2)) to account for differences in leg lengths or growth between visits. The GDI is a summary measure of an individual’s deviation from a TD control population for nine kinematic joint angles (pelvis: flexion/extension, internal/external rotation, adduction/abduction; hip: flexion/extension, internal/external rotation, adduction/abduction; knee: flexion/extension; and ankle: dorsiflexion/plantarflexion, foot progression angle) [40]. Similar to Walk-DMC, GDI is a scaled z-score such that the average of the clinic’s control kinematic database is 100, and every standard deviation from the average is represented by a 10-point decrease. Note that the clinic’s control kinematic database (*n* = 55, age: 10 + 7 (3 + 11) y + mo, mass: 40.0 (17.7) kg, height: 1.48 (0.21) m) is separate from the TD group with EMG data available that was used for comparing synergies. To align results with the standards of the clinic and use the full set of TD kinematics, we used the separate databases for these analyses. However, we did compare the databases and found the kinematics were similar and did not cause significant changes in the reported kinematic results.

### Statistical analyses

Descriptive statistics included the calculation of the average and standard deviation for synergy and gait metrics. One-way analysis of variance (ANOVA) with t-test post hoc were used to evaluate differences between groups pre-treatment on all continuous measures (tVAF_1_, synergy weights, synergy activations, GDI, and walking speed) [38]. A Kruskal-Wallis with rank-sum post-hoc was used to evaluate differences between groups pre-treatment on the ordinal measure, N_90_ [38]. Paired t-tests (for continuous data) and a Wilcoxon signed-rank test (for ordinal data) were used to evaluate changes between pre- and post-treatment [38]. To adjust for multiple comparisons in this study a Benjamini-Hochberg multiple comparison correction was applied to α = 0.05 [41].

To determine whether changes in synergies were associated with changes in gait post-treatment, we performed stepwise linear regressions for each outcome measure (e.g., speed and GDI). Stepwise regression started with a constant model, and regressors were added such that the sum of squared errors was minimized using an F-statistic at an alpha of 0.05 and critical *p* < 0.05. Initial potential regressors were pre-treatment GDI or walking speed, age, treatment group, and changes in synergies. These were chosen based on previous research suggesting their importance in gait outcomes [18]. Changes in synergies were measured with (1) tVAF_1_, (2) changes in synergy weights relative to the TD archetype, and (3) changes in synergy activations relative to the TD archetype. The model identified by the stepwise regression was recomputed with robust fitting using a bi-square weighting algorithm to minimize the effect of outliers in our regressions [42]. The impact of each regressor was assessed using effect sizes. Effect sizes were estimated from the adjusted response, computed by allowing each regressor to vary after averaging out the effects of the other regressors.

Model robustness was examined by performing a 10-fold cross-validation and comparing the resultant errors to the original model errors. Cross-validation was performed by replicating the regressions 10 times with 90% of the data and testing the resultant model on the withheld 10%, where each observation appears in a test set exactly once [43].

## Results

### Synergy complexity

**Fig. 1:**
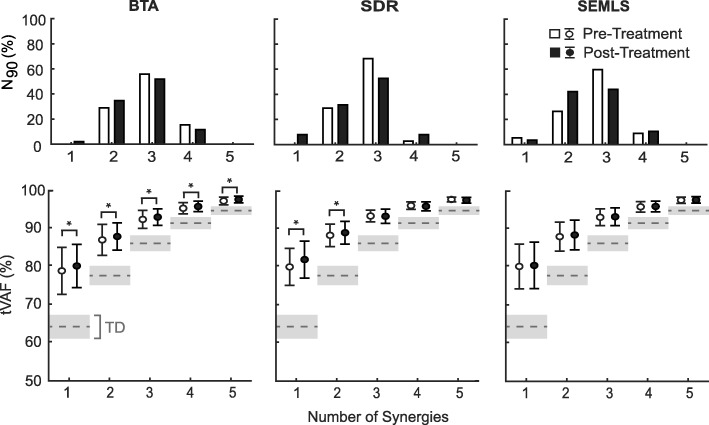
(Top) Histogram of the number of synergies to account for greater than 90% of the variance in EMG data (N_90_) for the children with CP (pre-treatment and post-treatment). (Bottom) Average (+/− 1 SD) total variance accounted for (tVAF) by one to five synergies for the children with CP (pre-treatment and post-treatment). The TD tVAF is shown in grey (average +/− 1 SD) for comparrison. *indicates significant change in tVAF_n_ following treatment (*p* < 0.05). *BTA* Botulinum Toxin Injection Type-A, *SDR* Selective Dorsal Rhysotomy, *SEMLS*, Single Event Multi-Level Orthopaedic Surgery, *TD* Typically-Developing Children

There were no significant differences in number of synergies (N_90_) pre-treatment between groups (*p* = 0.60) and N_90_ did not change significantly post-treatment for any treatment group (*p* > 0.10 for all groups). Similar to prior research, N_90_ was significantly smaller in the children with CP pre-treatment (average (SD): 2.78 (0.64)) compared to TD peers (4.19 (0.40), *p* < 0.001, Fig. 1). Number of synergies did change for some children: N_90_ changed for 33%, 40%, and 49% of individuals in the BTA, SDR, and SEMLS treatment groups, respectively. However, these changes were variable: 10% (BTA), 13% (SDR), and 18% (SEMLS) had an increase in N_90_, while 23% (BTA), 26% (SDR), and 32% (SEMLS) had a decrease in N_90_.

The total variance accounted for by a single synergy did not change for the SEMLS group (+ 0.3%, *p* = 0.69), but tVAF_1_ had a small, but significant change after BTA (+ 1.3%, *p* = 0.005) and SDR (+ 1.9%, *p* < 0.001, Fig. 1). Note in both cases tVAF_1_ increased, indicating that synergy complexity was further from TD peers post-treatment. Changes in tVAF_1_ corresponded to a 0.9, 4.1, and 6.2 point decreases in Walk-DMC for SEMLS, BTA, and SDR groups, respectively. The average (SD) tVAF_1_ pre-treatment was 79.1% (6.2%) for BTA, 80.1% (4.9%) for SDR, and 80.2% (5.9%) for SEMLS, which were all significantly greater than the average tVAF_1_ for the TD group of 64.4% (3.1%) (*p* < 0.001, Fig. 1). There was no significant difference in tVAF_1_ between groups pre-treatment (*p* = 0.46).

### Synergy composition

**Fig. 2:**
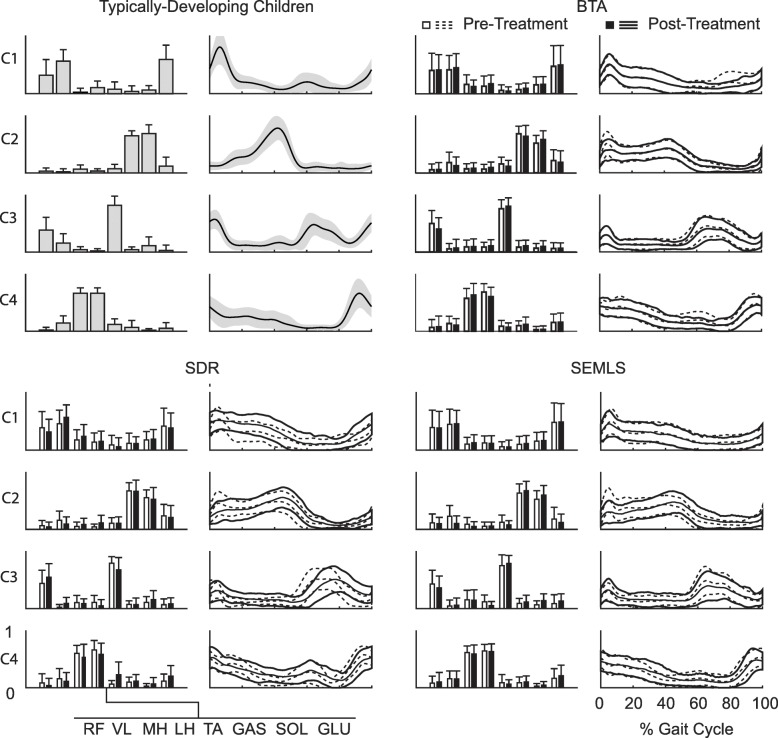
(Top Left) Average (± SD) synergy weights and activations for the typically developing children. Average TD weights and activations define the synergy archetypes that were used to compare synergies before and after treatment for the children with CP. Comparison of the average (± SD) pre- and post-treatment synergy weights and activations for BTA (Top Right), SDR (Bottom Left), and SEMLS (Bottom Right). *BTA* Botulinum Toxin Injection Type-A, *SDR* Selective Dorsal Rhysotomy, *SEMLS* Single Event Multi-Level Orthopaedic Surgery, *TD* Typically-Developing Children, *RF* Rectus Femoris, *VL* Vastus Lateralis, *MH* Medial Hamstrings, *LH* Lateral Hamstrings, *TA* Tibialis Anterior, *GAS* Medial Gasterocnemius, *SOL* Soleus, *GLU* Gluteus Medius

Synergy weights did not change significantly post-treatment (Fig. 2). The average similarity of the CP synergy weights to the TD archetypes pre-treatment were 0.77 (0.17), 0.88 (0.11), 0.90 (0.07), and 0.92 (0.10) for C1, C2, C3, and C4, respectively, and were not different between treatment groups (*p* = 0.73). After treatment, the average change in similarity to the TD synergy weights was 0.01 (0.08), −0.03 (0.14), and 0.02 (0.10) for the BTA, SDR and SEMLS groups, respectively and not statistically significant (*p* > 0.10 for all groups).

Synergy activations also did not change significantly after BTA or SEMLS, but there was a significant decrease in similarity to TD synergy activations after SDR. The average cosine similarity to the TD archetypes was similar between treatment groups pre-treatment (*p* = 0.08) and was 0.81 (0.12), 0.81 (0.09), 0.82 (0.07), and 0.86 (0.07) for C1, C2, C3, and C4, respectively. After treatment the average change in synergy activations was not significant at 0.01 (0.05) and − 0.01 (0.09) for the BTA and SEMLS groups, respectively, but was statistically significant at − 0.03 (0.07) for the SDR group (*p* = 0.01).

### Changes in gait

There were significant improvements in gait kinematics (Table 2) following SEMLS (pre/post GDI = 66/77, *p* < 0.001), but smaller changes after SDR (74/77, *p* = 0.06) and BTA (74/75, *p* = 0.91). After treatment 23%, 32%, and 67% of BTA, SDR, and SEMLS children increased their GDI scores by more than 5 points (minimum clinically significant difference, [44]), while 37%, 11%, and 5% decreased by more than 5 points, respectively. There were significant decreases in walking speed after SEMLS (0.29/0.24, *p* < 0.001) but smaller changes after BTA (0.32/0.30, *p* = 0.08) and SDR (0.34/0.30, *p* = 0.03, non-significant after multiple comparison correction). After treatment 15%, 21%, and 25% of the BTA, SDR, and SEMLS groups increased their dimensionless walking speed by more than 10% (clinically significant difference, [45]), while 50%, 42%, and 53% decreased by more than 10%, respectively.

**Table 2:** Participant outcomes

Treatment	N	Speed		GDI		N_90_		tVAF_1_	
		Pre	Post	Pre	Post	Pre	Post	Pre	Post
BTA	52	0.32 (0.14)	0.30 (.015)	74.4 (12.2)	74.6 (11.2)	2.87 (0.66)	2.73 (0.69)	0.79 (0.06)	0.80 (0.06)
SDR	38	0.34 (0.12)	0.30 (.011)	73.8 (10.2)	76.6 (13.1)	2.74 (0.50)	2.61 (0.75)	0.80 (0.05)	0.82 (0.05)
SEMLS	57	0.29 (0.11)	0.24 (.013)	66.4 (11.7)	76.8 (12.2)	2.72 (0.70)	2.61 (0.73)	0.80 (0.06)	0.80 (0.06)
TD	31	0.50 (0.09)	–	93.6 (9.3)	–	4.19 (0.40)	–	0.64 (0.03)	–

NOTE. Values are average (1 SD) or as otherwise indicated

*N* Number of Participants, *Post* Post-Treatment, *Pre* Pre-Treatment,

Speed Non-Dimensional Walking Speed, *GDI* Gait Deviation Index, N_90_ Number of Synergies,

*tVAF*_*1*_ Total Variance Accounted for By One Synergy, *BTA* Botulinum Toxin Type A Injection,

*SDR* Selective Dorsal Rhizotomy, *SEMLS* Single Event Multi-Level Orthopaedic Surgery,

*TD* Typically-Developing Children

**Fig. 3:**
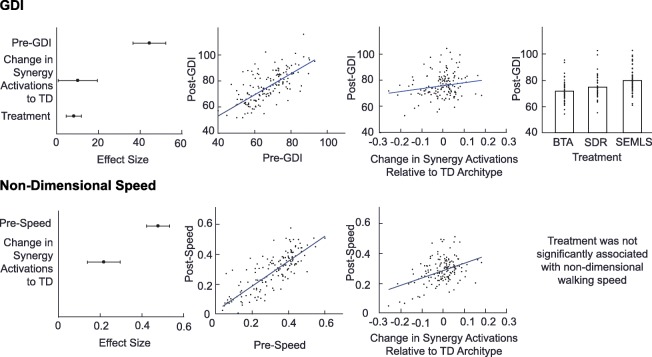
Effect size and adjusted response plots of significant regressors for post-treatment GDI and walking speed identified from stepwise regression. The estimated effect sizes and 95% confidence interval show which regressors are present in each model. Adjusted response plots show the relation between each outcome measure (post-treatment GDI or non-dimensional walking speed) and each predictor after removing the effect of the other predictors. Synergy activations that became closer to the TD archetypes were associated with better kinematics and faster walking speeds post-treatment. *BTA* Botulinum Toxin Injection Type-A, *GDI* Gait Deviation Index, *SDR* Selective Dorsal Rhysotomy, *SEMLS* Single Event Multi-Level Surgery, *TD* Typically Developing

Changes in gait kinematics and walking speed after treatment were significantly associated with changes in synergy activations (Table 3, Fig. 3), such that individuals whose synergy activations were more similar to TD peers after treatment had better outcomes. Neither changes in tVAF_1_ nor synergy weights were associated with changes in GDI or walking speed post-treatment. The average cross-validated model errors were less than 3% higher than the original model for GDI and within 1% of the original model for walking speed.

**Table 3:** Regression models of post-treatment GDI and walking speed

	Speed (r^2^ = 0.70)			GDI (r^2^ = 0.50)		
Term	Estimate	Standard Error	*p*	Estimate	Standard Error	*p*
Intercept^a^	0.02	0.02	0.16	–	–	–
				BTA: 21.33	4.92	<.001
				SDR: 24.16	5.01	<.001
				SEMLS: 29.28	4.42	<.001
Pre-Treatment	0.83	0.05	<.001	0.71	0.06	<.001
Change in Synergy Activations	0.49	0.09	<.001	22.27	10.50	0.036

^a^Treatment effect only for GDI

*GDI* Gait Deviation Index, *Speed* Non-Dimensional Walking Speed,

*BTA* Botulinum Toxin Type-A Injection, *SDR* Selective Dorsal Rhizotomy,

*SEMLS* Single Event Multi-Level Orthopaedic Surgery

## Discussion

Treatments for children with CP are often assumed to make dramatic changes to an individual’s musculoskeletal and neuromuscular systems. SEMLS and other orthopaedic surgeries alter the musculoskeletal system, reorienting bones, altering muscles paths, or lengthening tendons. BTA injections temporarily block muscle action potentials. SDR permanently removes some afferent feedback. After all of these treatments, children also receive extensive rehabilitation. While these treatments can induce significant changes in movement, our findings suggest that they have minimal impact on the underlying strategies that an individual uses to control and coordinate their muscles, suggesting that motor control is relatively fixed in CP.

While research has consistently demonstrated that individuals with neurologic injuries use a simplified control strategy compared to unimpaired individuals during locomotion [5, 12, 24, 26, 46], we found minimal changes in synergies after treatment. Although there was a small, but significant, increase in tVAF_1_ for BTA and SDR treatment groups, this change was in the opposite direction than desired: tVAF_1_ increased, creating a larger gap between the children with CP and TD peers. Both BTA and SDR treatments block or inhibit signals in the nervous system, potentially explaining this reduction in synergy complexity. In prior conference proceedings, van der Krogt and colleagues (2016) similarly reported a trend toward increasing tVAF_1_ after BTA, while Oudenhoven (2016) found no significant changes in tVAF_1_ following SDR. In all cases, the average change in tVAF_1_ has been less than 2%, suggesting minimal changes after treatment in CP [27, 28]. Moreover, a post-hoc analysis of the data found an average range in tVAF_1_ of 2.8% between trials within a session, roughly 1.5 times larger than the changes after SDR. Number of synergies (N_90_) demonstrated a similar trend of minimal changes. Although N_90_ changed after treatment for 41% of individuals, there were no significant changes for any treatment group. Rather these changes demonstrate that the number of synergies, an ordinal measure, may be inappropriate to evaluate changes in synergy complexity. For example, if an individual has a tVAF_n_ of 89% at one visit and 90% at another visit, their number of synergies would change despite only a small change in tVAF_n_. While both measures suggest minimal changes in synergy complexity after treatment in CP, we prefer to use tVAF versus N_90_ for greater granularity.

Synergy weights did not change after treatment, suggesting that similar groups of muscles were activated together. Synergy activations did change after SDR only, but again they were less similar to TD peers. Across all treatments, improvements in gait after treatment were only associated with changes in synergy activation that became more similar to TD peers. These findings highlight that even if coordination (i.e.*,* which muscles are being activated together) stays constant after treatment, changing patterns of recruitment (i.e.*,* synergy activations) can lead to improvements in gait. The importance of synergy activations was also demonstrated by Routson and colleagues (2013), who found that synergy activations, especially plantarflexor timing (synergy C2), were associated with improvements in kinematics and walking speed.

The lack of changes in synergy composition contrasts with research in unimpaired adults, where highly trained individuals have been found to have altered synergies compared to novices [47–49]. Further, interventions such as powered exoskeletons have been shown to alter synergy weights and activations [50–52]. Whether future innovations in treatments such as feedback training [50, 53, 54], forced exploration of new movement patterns [55], or electrical stimulation of the spinal cord [56] can induce similar changes in synergies for individuals with CP remains unknown. However, children with CP have been shown to have synergies more similar to neonates or toddlers [4, 37], and the altered maturation process of the brain and descending pathways may limit neural plasticity [57]. A reduction in neural plasticity could explain the small changes in synergies observed in this study even after drastic surgeries and extensive rehabilitation. Understanding the plasticity and impacts of treatments specifically targeted at neural control represent an important area of future research in CP.

As a retrospective study, this research was limited by clinical protocols. Children in this study walked without assistive devices when possible, but we did not exclude children who used them. However, walkers and other assistive devices can alter biomechanics and muscle activity [58–60], and understanding the impact of assistance on synergy complexity and structure represents an important area for future research. Although synergies have been shown to be repeatable between days for both TD and CP individuals [3, 61], the amount of time before and after treatment varied. Participants received therapy per their individual treatment plans as part of the standard of care. Thus, observed changes in synergies are due to the treatments analyzed in this study, along with a combination of rehabilitation [24–26], growth, and development [4, 37]. While the EMG data used to analyze synergies included the large muscles commonly targeted with treatment, it is possible that there are greater changes in activations or synergies for muscles not evaluated with EMG recordings as part of standard clinical gait analysis. Similarly, the amount and quality of data varied between individuals and sessions. Prior research has shown that number of gait cycles can impact synergies, especially for small numbers of gait cycles [30]. Thus, we chose to use all available trials in our analysis, accounting for as much variability between gait cycles as possible. Missing data in some individuals necessitated the use of WNMF to calculate synergies, which could cause some changes in the synergy outputs. A post-hoc comparison between synergies calculated using the WNMF algorithm on sessions with complete data and the same sessions where data was omitted (up to 70% of one EMG channel and 30% of a second EMG channel, with non-overlapping portions) found an average change in tVAF_n_ of < 1% for *n* = 1–5 synergies and an average cosine similarity > 0.95 for synergy weights and activations.

## Conclusions

This study demonstrated that common treatments in CP, including extensive rehabilitation, resulted in minimal changes in muscle synergies. There were decreases in synergy complexity after BTA and SDR, but these changes were small and resulted in synergy complexity less similar to TD peers. Changes after treatment were variable across participants, emphasizing the heterogeneity of movement patterns in CP that necessitate better methods to quantify patient-specific differences in motor control and movement. Across treatments, changes in synergy activations were associated with changes in gait. Children whose synergy activations were more similar to TD peers after treatment had greater improvements in kinematics and walking speed. These results highlight that, although synergy complexity and weights are challenging to change in CP, synergy activations may provide a target for rehabilitation to improve gait.

## Abbreviations

ANOVA: Analysis of variance
BTA: Botulinum toxin type-A injections
CP: Cerebral palsy
EMG: Electromyography
GDI: Gait deviation index
GMFCS: Gross motor function classification System
N_90_: Number of synergies to account for 90% of the variance in the EMG data
NMF: Non-negative matrix factorization
SDR: Selective dorsal rhizotomy
SEMLS: Single event multi-level orthopaedic surgery
TD: Typically-developing Children
tVAF_n_: Total variance accounted for by n synergies
Walk-DMC: Dynamic motor control index during walking
WNMF: Weighted non-negative matrix factorization
